# Association Between Unequal Division of Caregiving Work and South Korean Married Women's Depressive Symptoms

**DOI:** 10.3389/fpubh.2022.739477

**Published:** 2022-03-29

**Authors:** Minah Park, Jieun Jang, Hye Jin Joo, Gyu Ri Kim, Eun-Cheol Park

**Affiliations:** ^1^Department of Public Health, Graduate School, Yonsei University, Seoul, South Korea; ^2^Institute of Health Services Research, Yonsei University, Seoul, South Korea; ^3^Hinda and Arthur Marcus Institute for Aging Research, Hebrew SeniorLife, Harvard Medical School, Boston, MA, United States; ^4^Department of Hospital Administration, Yonsei University Graduate School of Public Health, Seoul, South Korea; ^5^Department of Preventive Medicine, Yonsei University College of Medicine, Seoul, South Korea

**Keywords:** caregiving, depressive symptoms, unpaid care work, gender equality, married women, South Korea

## Abstract

**Background:**

A disproportionate amount of family caregiving can negatively impact married women's mental health. This study aims to examine the relationship between depressive symptoms in South Korean women and the satisfaction with their husband's participation in family caregiving.

**Methods:**

Raw data from 1,515 of the participants in the 2014, 2016, and 2018 Korean Longitudinal Survey of Women and Families were analyzed. Satisfaction with husbands' participation in family caregiving was classified as satisfied, less satisfied, and not satisfied. The survey used the Center for Epidemiologic Studies Depression Scale to measure depressive symptoms. The association was examined using a generalized estimating equations model.

**Results:**

Results indicated 22.2% of the participating women reported depressive symptoms. Women who reported dissatisfaction with their husband's participation in caregiving were 2.54 times more likely to report depressive symptoms than the women who were satisfied. Subgroup analysis indicated that women with higher levels of education, were more likely to have depressive symptoms when they were not satisfied with their husbands' participation in caregiving.

**Conclusion:**

Married women who reported being dissatisfied with their husbands' participation in caregiving were more likely to report depressive symptoms. These results suggest the need to create environments with fair distribution of caregiving duties to minimize depressive symptoms in women.

## Introduction

**Figure 1:**
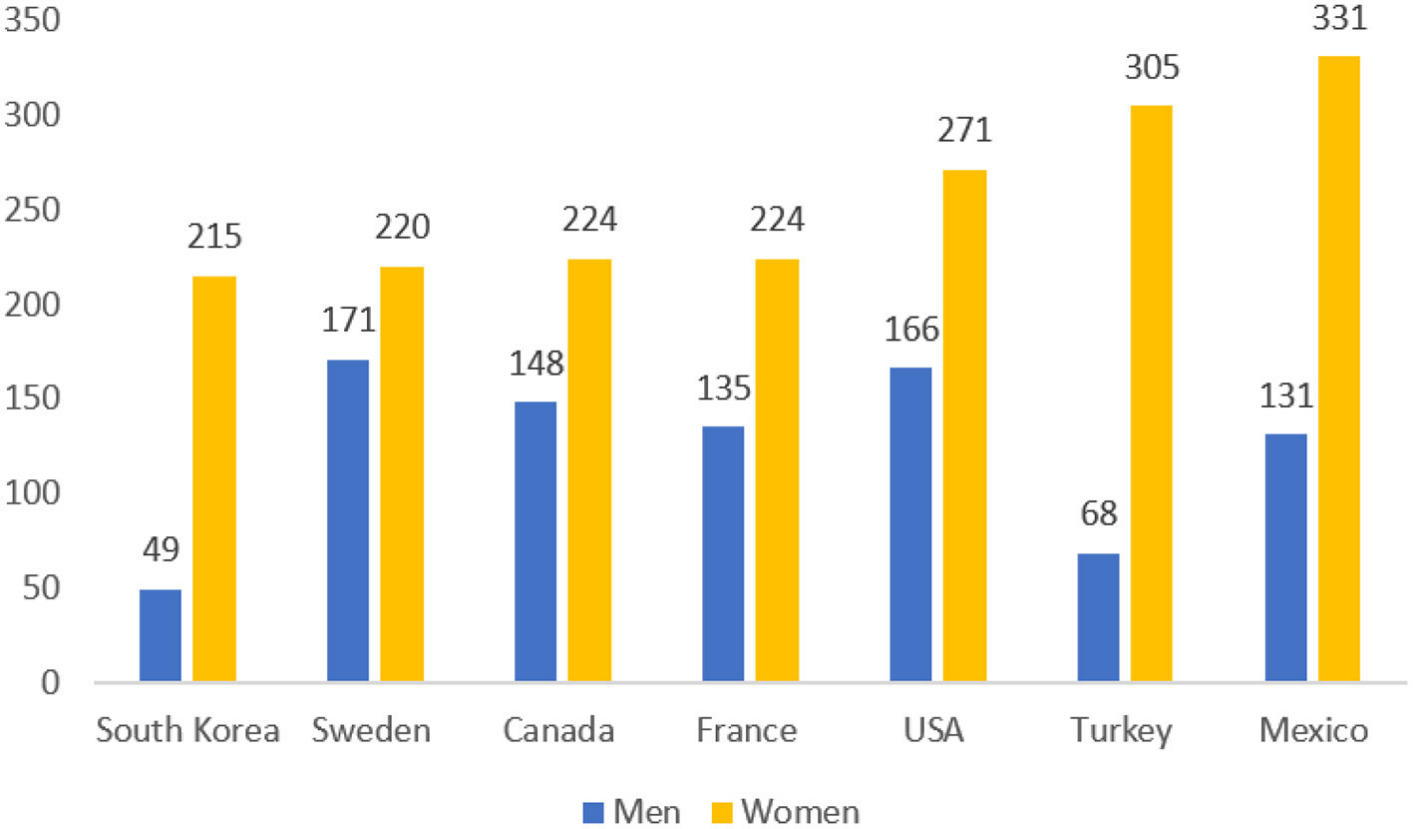
Average unpaid work (minutes) by gender in major countries (Source: Statistics Korea).

Unpaid care work is defined as all unpaid services provided within a household, including taking care of persons, housework, and the like (1). Such work constitutes an indispensable factor contributing to the wellbeing of individuals, families, and society (2). Women typically spend a larger proportion of time on unpaid care work than men. This applies to women across all socio-economic levels, cultures, and regions. According to the Organisation for Economic Co-operation and Development, the average number of hours spent worldwide on unpaid care work was much higher for women than for men (Figure 1) (3). In South Korea, the gender gap for unpaid care work is much higher than that in many other countries. Reportedly, women spend an average amount of 54 min per day on the unpaid care of children or elderly people, and men spend 17 min per day (4).

South Korea has a strong family orientation, and women have traditionally been assigned the primary responsibility to care for children and the elderly (5). However, the change in this social climate has catalyzed a change in the family roles of men, promoting the need for joint participation and responsibility between spouses toward raising a family. A prime example of this change is seen in media, with male celebrities and their children normalizing fatherhood and parenting through television appearances; and in new buzzwords such as “Latte Papa” (sophisticated dads who hang out with their kids in coffee shops) (6).

Despite these social changes, women still play a large role in domestic work, and the weight of this role is known to be burdensome and stressful (7). This is seen to negatively contribute to their quality of life, increase problems in the family, and lead to deteriorated mental health (8). An increased number of women graduating from tertiary education and entering the workforce has led to an increase in dual-income couples and equality among families regarding unpaid work, especially where caregiving is needed.

Numerous studies have examined many aspects of married women's mental health. For example, Lee et al. (9) examined the association between women's satisfaction with their husbands' participation in housework and suicidal ideation. Jung et al. (10) also found an association between equitable division of housework and women's depression. However, these studies are limited in that they only consider housework as unpaid care, while disregarding caregiving as an aspect. This study aims to fill this gap in the literature, by analyzing the relationship between depressive symptoms in South Korean women and the satisfaction with their husband's participation in family caregiving, using nationally representative data. To the best of our knowledge, this is the first study to investigate this association using the data described below.

## Methods

### Data

The study relies on self-reported data collected using the Korean Longitudinal Survey of Women and Families (KLoWF), by the Korea Women's Development Institute (KWDI) in 2014, 2016, and 2018. The KWDI is a government think-tank under the Prime Minister's Office of South Korea since 1982 and has dedicated itself to research gender and family issues in South Korea (11). The KLoWf is a panel survey that was first launched in 2007 to analyze the effectiveness of female-oriented policies (12). The data used stratification and multistage sampling design using Korean Population and Housing Census data to ensure a representative sample. The survey collected data using the Computer Assisted Personal Interviewing (CAPI) (13) method. The KLoWF includes basic questions about the household, such as income level and type of housing; family-values-related questions; questions about personal experiences, such as marital life, family relations, and health; and questions about working conditions, such as job satisfaction and maternity leave.

### Participants

The total participants included 7,745 women at the initial baseline of 2014. Data from participants who did not fit the criteria were excluded. This included unmarried women (*n* = 1,051), divorcees (*n* = 252), separated (*n* = 42), widowed (*n* = 645), those who are married but do not have the responsibility of caregiving (*n* = 3,805) and those for whom variables were missing (*n* = 435). Data from 1,515 participants were ultimately analyzed. The KLoWF obtained participant's informed consent during the CAPI, and the data were made available to the public, with private information excluded. The study did not require prior approval from the Institutional Review Board or informed consent since the KLoWF is a secondary dataset available in the public domain, and does not contain private information. The microdata (in the form of Statistical Analysis Software [SAS] files) and analytic guidelines can be downloaded from the KLoWF website (https://klowf.kwdi.re.kr/portal/eng/mainPage.do).

### Variables

The variable of interest in the present study was the level of satisfaction with husbands' participation in caregiving, which the following question in the KLoWF measured: “How satisfied are you with your husband's degree of participation in caregiving activities such as taking care of children or taking care of older parents?” The five-point answer scale ranges from “very satisfied” to “very unsatisfied.” We simplified the responses into “satisfied” (includes “very satisfied,” and “satisfied”), “less satisfied” (includes “averagely satisfied”), and “not satisfied” (includes “dissatisfied” and “not satisfied at all”).

The dependent variable, depressive symptoms, was measured by the Center for Epidemiologic Studies Depression Scale (CES-D-10). First used in the late 1970's, the CES-D-10 has become known for its reliability and validity in the context of both primary care patients and the general population (14). It comprises 10 questions: questions 5 and 8 measure depression based on positive symptoms, and the other questions measure depression based on negative symptoms. All items are assessed on a four-point scale (0–3). As is standard, this study categorizes participants with scores of 10 or higher as depressed.

The independent variables included age, working status, education level, husband's education level, husband's occupation, satisfaction with husband's participation in housework, outside help with domestic work, presence of parents and in-laws in the home, marital satisfaction, household income, presence of children (under the age of 18), and subjective health conditions.

### Statistical Analysis

Chi-squared test and analysis of variance were used to summarize the general characteristics of the study population. We also evaluated the relationship between women's satisfaction with their husbands' participation in caregiving and the women's depressive symptoms using a generalized estimating equation (GEE) model. The GEE model is used in longitudinal studies or repeated measure research designs with non-normal responses and has been known to be efficient, providing unbiased regression estimates (15). In our study, those who participated only in one of the years were measured once, those who participated in two of the years were measured twice, and those who participated in all three surveys conducted in 2014, 2016, and 2018 were measured thrice. Subgroup analyses were performed separately after controlling the covariates. Results were reported using odds ratios (ORs) and confidence intervals (CIs). The data analysis was conducted with SAS 9.4 (SAS Institute Inc; Cary, North Carolina).

## Results

Table 1 presents the general characteristics of the study participants in the baseline year. Overall, 22.2% of the participants in the present study scored 10 or higher on the CES-D-10. The more dissatisfied a woman was with her husband's participation in caregiving, the higher she scored on the CES-D-10. Among women who were satisfied with their husbands' participation, 16.3% reported depressive symptoms. Among women who were less satisfied, 23.7% reported depressive symptoms. Among women who were not satisfied, 33.7% reported being depressed. Moreover, those who came from low income households (37.6%), had no satisfaction with their husband's participation in housework (34.5%), and had low marital satisfaction (38.9%) also reported being depressed.

**Table 1:** General characteristics of study subjects.

	**CESD-10 ≥ 10**						
	**Total**		**Yes**		**No**		
	***N***	**%**	***N***	**%**	***N***	**%**	***P*** **-Value**
**Total(*****n*** **= 1,515)**	**1,515**	**(100)**	**336**	**(22.2)**	**1,179**	**(77.8)**	
Satisfaction with husband's participation in caregiving							<0.0001
Satisfied	656	(43.3)	107	(16.3)	549	(83.7)	
Less satisfied	607	(40.1)	144	(23.7)	463	(76.3)	
Not satisfied	252	(16.6)	85	(33.7)	167	(66.3)	
Age group							<0.0001
20–39	637	(42.0)	118	(18.5)	519	(81.5)	
40–59	684	(45.1)	144	(21.1)	540	(78.9)	
60 and over	194	(12.8)	74	(38.1)	120	(61.9)	
Educational level							0.0014
High School or Lower	713	(47.1)	184	(25.8)	529	(74.2)	
University or Higher	802	(52.9)	152	(19.0)	650	(81.0)	
Working status							0.1259
Yes	714	(47.1)	146	(20.4)	568	(79.6)	
No	801	(52.9)	190	(23.7)	611	(76.3)	
Household income							<0.0001
Low	178	(11.7)	67	(37.6)	111	(62.4)	
Low-Middle	525	(34.7)	134	(25.5)	391	(74.5)	
Middle-High	483	(31.9)	92	(19.0)	391	(81.0)	
High	329	(21.7)	43	(13.1)	286	(86.9)	
Children (under 18)							0.0001
None	366	(24.2)	109	(29.8)	257	(70.2)	
Only preschool	336	(22.2)	53	(15.8)	283	(84.2)	
Only school	493	(32.5)	102	(20.7)	391	(79.3)	
Both preschool and school	320	(21.1)	72	(22.5)	248	(77.5)	
Husband age							0.0032
20–39	172	(11.4)	26	(15.1)	146	(84.9)	
40–59	260	(17.2)	62	(23.8)	198	(76.2)	
60 and over	73	(4.8)	25	(34.2)	48	(65.8)	
Husband education level							0.2098
High school or lower	902	(59.5)	210	(23.3)	692	(76.7)	
University or higher	613	(40.5)	126	(20.6)	487	(79.4)	
Husband occupation							<0.0001
White	520	(34.3)	91	(17.5)	429	(82.5)	
Blue	255	(16.8)	59	(23.1)	196	(76.9)	
Pink	506	(33.4)	107	(21.1)	399	(78.9)	
None	234	(15.4)	79	(33.8)	155	(66.2)	
Satisfaction with husband's participation in housework							<0.0001
Satisfaction	651	(43.0)	114	(17.5)	537	(82.5)	
Less satisfaction	597	(39.4)	130	(21.8)	467	(78.2)	
No satisfaction	267	(17.6)	92	(34.5)	175	(65.5)	
Help for domestic housework							0.0012
Yes	103	(6.8)	36	(35.0)	67	(65.0)	
No	1,412	(93.2)	300	(21.2)	1,112	(78.8)	
Surviving parent							<0.0001
Yes	1,198	(79.1)	224	(18.7)	974	(81.3)	
No	317	(20.9)	112	(35.3)	205	(64.7)	
Surviving in-law							<0.0001
Yes	1,133	(74.8)	223	(19.7)	910	(80.3)	
No	382	(25.2)	113	(29.6)	269	(70.4)	
Marriage satisfaction							<0.0001
Good	1,222	(80.7)	222	(18.2)	1000	(81.8)	
Bad	293	(19.3)	114	(38.9)	179	(61.1)	
Subjective health condition							<0.0001
Good	1,009	(66.6)	165	(16.4)	844	(83.6)	
Normal	398	(26.3)	107	(26.9)	291	(73.1)	
Bad	108	(7.1)	64	(59.3)	44	(40.7)	

Table 2 presents the results of the GEE model analyses. Women who were less satisfied with their husbands' participation were 1.70 times more likely to report depressive symptoms (OR: 1.70, CI: 1.02–2.83). Women who were not satisfied with their husbands' participation were 2.54 times more likely to report depressive symptoms (OR: 2.54, CI: 1.37–4.69). Moreover, when their levels of marital satisfaction were low, the participating women were 1.82 times more likely to report depressive symptoms (OR: 1.80, CI: 1.25–2.66). Moreover, compared to those with good health condition, those having normal and bad health conditions were 1.60 times and 4.79 times more likely to report depressive symptoms, respectively (OR: 1.60, CI: 1.05–2.42, OR: 4.79, CI: 2.51–9.17).

**Table 2:** Associations between Depressive symptoms (CES-D-10) and subject demographics.

**Variables**	**(CESD ≥ 10)**	
	**OR**	**95% CI**
Satisfaction with husband's participation in caregiving		
Satisfied	1.00	
Less satisfied	1.70	(1.02 - 2.83)
Not satisfied	2.54	(1.37 - 4.69)
Age group		
20–39	1.00	
40–59	0.68	(0.41 - 1.11)
60 and over	0.80	(0.34 - 1.88)
Educational level		
High school	1.03	(0.64 - 1.65)
University or higher	1.00	
Working status		
Yes	1.00	
No	0.87	(0.59 - 1.28)
Household income		
Low	1.64	(0.75 - 3.61)
Low-middle	2.17	(1.26 - 3.74)
Middle-high	1.78	(1.03 - 3.08)
High	1.00	
Children (under 18)		
None	1.00	
Preschool age	0.90	(0.39 - 2.09)
School age	1.31	(0.69 - 2.52)
Both preschool and school age	1.36	(0.63 - 2.95)
Husband education level		
High school or lower	0.70	(0.44 - 1.11)
University or higher	1.00	
Husband occupation		
White	1.00	
Blue	1.09	(0.60 - 1.98)
Pink	1.04	(0.59 - 1.85)
None	1.36	(0.73 - 2.55)
Satisfaction with husband's participation in housework		
Satisfaction	1.00	
Less satisfaction	0.96	(0.58 - 1.59)
No satisfaction	1.12	(0.59 - 2.10)
Help for domestic housework		
Yes	1.00	
No	0.40	(0.20 - 0.81)
Surviving parent		
Yes	0.67	(0.35 - 1.27)
No	1.00	
Surviving in-law		
Yes	0.88	(0.52 - 1.47)
No	1.00	
Marriage satisfaction		
Good	1.00
Bad	1.82	(1.25 - 2.66)
Subjective health condition		
Good	1.00	
Normal	1.60	(1.05 - 2.42)
Bad	4.79	(2.51 - 9.17)

Table 3 presents the results of analyses of the association between the variables in the subgroup of satisfaction with husbands' participation in caregiving and the women's depressive symptoms. Women with higher education levels when not satisfied with their husbands' caregiving participation (OR: 3.44, CI: 1.47–8.07) and working women who were not satisfied with their husbands' caregiving participation had a higher risk for depressive symptoms (OR: 3.29, CI: 1.39–7.77). Furthermore, women in families with no help for domestic housework and women who were less satisfied or not satisfied with their husbands' caregiving efforts were at higher risk for depressive symptoms (OR: 2.36, CI: 1.40–3.99; OR: 2.89, CI: 1.55–5.38). Finally, low marital satisfaction along with dissatisfaction with the husband's caregiving participation indicated a higher risk for depressive symptoms (OR: 4.97, CI 1.16–21.23).

**Table 3:** Subgroup analysis stratified by independent variables.

**Variables**	**CESD-10 ≥ 10**				
	**Satisfaction with husband's participation in caregiving**				
	**Satisfied**	**Less satisfied**		**Not satisfied**	
	**OR**	**OR**	**95% CI**	**OR**	**95% CI**
Educational level					
High School	1.00	1.45	(0.71 – 2.98)	2.43	(0.98 – 6.02)
University or Higher	1.00	2.52	(1.17 – 5.42)	3.44	(1.47 – 8.07)
Working status					
Yes	1.00	1.46	(0.72 – 3.00)	3.29	(1.39 – 7.77)
No	1.00	2.98	(1.41 – 6.28)	2.16	(0.84 – 5.56)
Help for domestic housework					
Yes	1.00	0.08	(0.02 – 0.44)	0.33	(0.01 – 14.27)
No	1.00	2.36	(1.40 – 3.99)	2.89	(1.55 – 5.38)
Marriage satisfaction					
Good	1.00	1.41	(0.72 – 2.37)	2.90	(1.19 – 7.08)
Bad	1.00	2.82	(0.70 – 11.37)	4.97	(1.16 – 21.23)

## Discussion

This study analyzed self-reported data from the KLoWF to examine associations between women's satisfaction with their husbands' participation in caregiving and experience with depressive symptoms. The present study highlights when the women were dissatisfied with their husbands' participation in caregiving, they were more likely to report depressive symptoms. Moreover, those with a higher education level, those who were not satisfied with their marriage, and those who were employed were more likely to be depressed.

Our results indicate that women who were dissatisfied with their husband's participation in caregiving were more likely to have depressive symptoms. The influence of culture has had a marked impact on women, who are expected to take on the burden of unpaid work. The intensive physical labor and strength required for caregiving labor is a major cause of burden leading to fatigue and stress in women (8). Stress can activate the release of hormones, including cortisol (16), a high level of which is known to have a deteriorating effect on mental health, including depression (17). Compared to men, women with higher level of objective stress also perceive their stress to be higher. (18). The unpaid work, lack of personal time for leisure and self-care, and lack of communication between partners could lead to emotional distresses such as anxiety and depression (19).

This result aligns with that of other studies that focus on unequal division of unpaid work. In a study from the US (20), there was a difference in depression between sexes due to inequalities in housework. Another study from the United Kingdom (21) showed that during the early stages of Covid-19, women ended up doing more than two-third of the household work and childcare. Increased childcare was also associated with psychological distress among women, when compared to men.

Women with higher levels of education were found to be at a higher risk for depressive symptoms when dissatisfied with their husbands' participation in caregiving. It is well known that highly educated women are likely to have a strong desire to establish an independent self-identity and self-actualize. For women who crave an independent self, the role of a mother is may not fully satisfying (22). Additionally, women with higher levels of education are more likely to have learned about gender equality and to regard it as an important factor. When their husbands' participation in caregiving is not equal to their own, these women are more likely to present with depressive symptoms.

Results also indicated that when marital satisfaction was low, the women were at a higher risk for depressive symptoms when dissatisfied with their husband's participation in caregiving. Women have been found to be relatively more vulnerable to and emotionally affected by problems that continue to arise in marital relationships than men. In particular, they reported feeling lonely and alienated when in unequal and emotionally distant relationships (23). Wives may view husbands' participation in caregiving work as an act of expressing support for them, suggesting that such participation can increase women's marriage satisfaction (24). Moreover, unhappy marriages are seen as a particularly serious risk to women's mental health.

Working women were at a higher risk for depressive symptoms when dissatisfied with their husband's participation in caregiving. This aligns with Roxburgh (25) finding that the stress of caregiving can lead to depression in working women. Another study found that despite both parties contributing to the family income, most of the family responsibility still falls to the wife, making a comfortable work–family balance difficult for her to achieve (26). According to a study on childcare, married women spend more time interacting—defined as one-on-one time, including feeding or bathing—with their children, whereas the husbands' participation with children was more passive such as watching television or simply playing with the children (27). This dynamic does not tend to change even when the husband works less than his wife (28). Notably, another study indicated that while married men's burnout is primarily influenced by their work, married women's burnout is more influenced by both family and work (29). Working wives are also likely to experience “motherhood penalty” at work, that is, being required to complete a full workload in less time due to the time spent on caregiving. When fathers are more involved, mothers tend to be less burdened at work because they have more time to complete their duties (30). Negative spillover between work and family, which is most frequently characterized by various types of work-family conflict or interference, has been linked with psychological distress and marital dissatisfaction (31). Conversely, when fathers are not involved in caregiving, mothers' workplace burdens are likely to be heavy, creating stress for the women and putting them at risk for depression (32).

The current study has several limitations. First, the results were obtained from a single self-report questionnaire posed to women about their husband's participation in housework, and therefore, are not likely to reflect husband's real caregiving time. Additionally, the KLoWF does not distinguish between child caregiving and elderly caregiving. Second, although the CES-D 10 has been tested for validity and reliability, the participant's self-reports of depressive symptoms are subjective and were not verified through objective measures. Third, the KloWF did not include other health factors such as smoking status, alcohol consumption, or exact health conditions that could affect depression.

However, the KLoWF, was conducted by the KWDI, a trustworthy national institution, using skilled interviewers and a computer-based system to obtain results directly from the participants. The CES-D-10 is an easy to administer and valid tool for screening patients with depressive symptoms. We do not, therefore, suspect that inaccurate self-reporting of depression in the CES-D 10 had a significant effect on the results of this study.

The strength of this study includes the data used to determine the association being obtained from the most recent nationally representative database, making the results obtained highly representative of the adult women population in South Korea. Finally, to our knowledge, this is the first study to determine the association between satisfaction with husbands' participation in caregiving and depressive symptoms using the CES-D-10 with KLoWF.

The results of this study confirm an association between satisfaction with husbands' participation in caregiving and married women's depressive symptoms. This indicates that gender equality in family caregiving is needed to improve women's mental health and promote a more equal society. Additional public polices such as equal maternity, paternity leave, and equal guaranteed leave for their children and elderly parents should be put in place.

## Data Availability Statement

Publicly available datasets were analyzed in this study. This data can be found in the KLoWF website https://klowf.kwdi.re.kr/portal/mainPage.do.

## Ethics Statement

Ethical review and approval was not required for the study on human participants in accordance with the local legislation and institutional requirements. The patients/participants provided their written informed consent to participate in this study.

## Author Contributions

MP designed this study, performed statistical analysis, drafted, and completed the manuscript. JJ, HJ, and GK contributed to the concept and design of the study and revised the manuscript. E-CP directed this study. All authors read and approved the final manuscript.

## Conflict of Interest

The authors declare that the research was conducted in the absence of any commercial or financial relationships that could be construed as a potential conflict of interest.

## Publisher's Note

All claims expressed in this article are solely those of the authors and do not necessarily represent those of their affiliated organizations, or those of the publisher, the editors and the reviewers. Any product that may be evaluated in this article, or claim that may be made by its manufacturer, is not guaranteed or endorsed by the publisher.
